# Supplementary material for: A responsivity theory of call timing patterns in bat echolocation
Source: BMC Biol. 2026 Oct 31;24:234. doi: 10.1186/s12915-026-02774-6 (PMC13753469; doi:10.1186/s12915-026-02774-6)
Supplement: Supplementary file 1 [file 12915_2026_2774_MOESM1_ESM.pdf]

# A responsivity theory of call timing patterns in bat echolocation

## Supplementary Information

Ravi Umadi [1,*] Uwe Firzlaff [1]

[1] Lehrstuhl für Zoologie, TUM School of Life Sciences, Technische Universität München (TUM), Liesel-Beckmann-Str. 4, 85354, Freising-Weihenstephan, Germany

*Corresponding author: `ravi.umadi@tum.de`

### CONTENTS

**S1 Notation and parameter glossary** 2

**S2 Conditional analytical reference** 4

**S3 Selected derivations and safeguards** 7
- S3.1 General inverse family . . . 7
- S3.2 First-arrival and terminal rearrangements . . . 7
- S3.3 Trajectory derivative . . . 7

**S4 Secondary numerical results** 8

### LIST OF TABLES

- S1 Core notation of the responsivity framework . . . 2
- S2 Simulation and variability notation . . . 3
- S3 Analytical lookup table for conditional calculations . . . 4
- S4 Assumption and identifiability map . . . 6
- S5 Echo-window effects on timing and call-rate spread . . . 8
- S6 Interval–distance fits across parameter sweeps . . . 8
- S7 Field–simulation statistics and conditional profiles . . . 9
- S8 Buzz-readiness summaries across parameter sweeps . . . 9
- S9 Proxy and time-to-contact comparisons for buzz readiness . . . 9
- S10 Structure of SSG-like events . . . 9

**Abbreviations:** CF, constant frequency; FM, frequency modulated; IPI, interpulse interval; IQR, interquartile range; MAD, median absolute deviation; SD, standard deviation; SSG, sonar strobe group; TTC, time to contact.
References to equations, figures, and sections without an S prefix refer to the accompanying main manuscript.

The supplementary material first provides a notation glossary and a conditional analytical reference for applying the responsivity framework. These are followed by selected derivations and the complete secondary numerical results supporting the streamlined accounts in the main text. Quantities described as recoverable below are conditional on the listed inputs and assumptions; none of the inverse identities resolves several unknown latent variables from call timing alone.

## S1 NOTATION AND PARAMETER GLOSSARY

**Table S1: Core notation of the responsivity framework.** "Observed" denotes quantities available from call timing or emitted signals; "measured" denotes quantities requiring spatial or kinematic observations; "latent" denotes quantities that can be inferred only after the required model parameters and anchor have been specified. All event-level quantities may vary with call index (n).

| Symbol | Definition | Units | Status and qualification |
|---|---|---|---|
| $n$ | Call-event index; interval $n$ begins at onset of call $n$ and ends at onset of call $n+1$. | – | Indexing convention. |
| $t_n^{\text{on}}, t_n^{\text{off}}$ | Onset and offset times of call $n$. | s | Observed from the emitted call. |
| $t_n^{\text{echo,start}}, t_n^{\text{echo,end}}$ | Arrival of the first relevant echo and behaviourally selected end of the acquired echo information. | s | Echo start may be measured if the return is reconstructed; echo end is generally latent and anchor-dependent. |
| $\mathrm{x}_{b,n}, \mathrm{x}_{q,n}$ | Bat and effective-anchor positions at call onset. | m | Require call-resolved localisation and anchor identification. |
| $\mathrm{r}_n, d_n, \hat{\mathrm{r}}_n$ | Bat-to-anchor displacement vector, its magnitude, and the corresponding unit vector. | m, m, – | Geometric quantities derived from the two positions. |
| $\mathrm{v}_{b,n}, \mathrm{v}_{q,n}$ | Bat and effective-anchor velocity vectors. | m s$^{-1}$ | Require trajectory measurements. |
| $\mathrm{v}_{\text{rel},n}$ | Anchor velocity relative to the bat, $\mathrm{v}_{q,n} - \mathrm{v}_{b,n}$. | m s$^{-1}$ | Derived from measured velocity vectors. |
| $v_{r,n}$ | Radial closing velocity, positive when bat–anchor range decreases. | m s$^{-1}$ | Measured from trajectories or conditionally inferred; it is not total flight speed. |
| $c$ | Speed of sound under the relevant atmospheric conditions. | m s$^{-1}$ | Known or measured environmental quantity. |
| $T_{c,n}$ | Emitted duration of call (n). | s | Directly observed. |
| $\Delta t_n$ | Onset-to-onset interpulse interval following call (n). | s | Directly observed; $\Delta t_n = 1/C_{r,n}$. |
| $S_n$ | Silent offset-to-next-onset interval, $\Delta t_n - T_{c,n}$. | s | Derived from observed call timing and duration. |
| $T_n^{\text{delay}}$ | Propagation delay from call onset to the first relevant echo. | s | Determined by anchor range and radial motion under the adopted propagation approximation. |
| $T_{\phi,n}$ | Selected echo-stream information window following the first relevant arrival. | s | Latent; parameterised here as $\phi_n T_{c,n}$. |
| $\phi_n$ | Dimensionless scaling of the call-linked echo-information window. | – | Latent/model parameter; $\phi = 0$ is first-arrival anchoring and values above one are not excluded by the formulation. |
| $T_{a,n}$ | Acoustic acquisition interval from call onset to the selected information endpoint. | s | Latent or conditionally inferred; $T_a = T^{\text{delay}} + T_\phi$. |
| $T_{e,n}$ | Offset-referenced time from call offset to the selected echo-information endpoint. | s | Derived constituent, $T_e = T_a - T_c$; not an additional interval in the IPI. |

*Continued on next page*

*Supplementary Table S1 continued*

| Symbol | Definition | Units | Status and qualification |
|---|---|---|---|
| $T_{b,n}$ | Post-acquisition behavioural interval before the next call. | s | Latent aggregate interval; not synonymous with a complete overt reaction time. |
| Conceptual $T_b$ components | $T_{\mathrm{aff}}$, $T_{\mathrm{sel}}$, $T_{\mathrm{dec}}$, $T_{\mathrm{mot}}$, and $T_{\mathrm{lag}}$: afferent, selection or integration, decision, motor, and voluntary or stochastic-lag contributions. | s | Illustrative components that may overlap physiologically; they are not separately estimated by the framework. |
| $k_r$ | Responsivity coefficient in $T_b = k_r T_a$. | – | Effective model parameter; must be specified or independently estimated before partitioning an observed IPI. |
| $C_{r,n}$ | Call rate, reciprocal of onset-to-onset IPI. | Hz | Directly derived from observed timing. |
| $\dot{C}_r$, $\dot{v}_r$ | Time derivatives of call rate and radial closing velocity. | Hz s$^{-1}$, m s$^{-2}$ | Trajectory quantities requiring smoothing or sufficiently resolved continuous estimates. |
| $K$ | Compact first-arrival factor, $K = 2(1 + k_r)$. | – | Defined abbreviation, not an independent parameter. |
| $L_{\mathrm{signal},n}$ | Spatial length occupied by a call, $cT_{c,n}$. | m | Derived from call duration. |
| $\Delta R_n$ | Range separation below which echoes overlap in time, $cT_{c,n}/2$. | m | Far-field acoustic-depth measure. |
| $d_{\mathrm{overlap},n}$ | Motion-aware range at which first-echo delay equals call duration. | m | Conditional on the relevant anchor and radial velocity. |
| $d_{\mathrm{stop}}$ | Specified stop, interception, or terminal reference distance. | m | Experimental or modelling boundary; not assumed universal across species or tasks. |
| $C_{r,\max}$ | Call-rate ceiling implied at $d_{\mathrm{stop}}$. | Hz | Theoretical ceiling under the stated anchoring assumptions; an observed maximum need not attain it. |
| $T_{a,\min}$, $T_{b,\min}$ | Acoustic and behavioural intervals realised at the specified terminal boundary or ceiling. | s | Boundary-dependent derived quantities, not universal physiological minima. |
| $f$ | Fraction of $C_{r,\max}$ used to define a terminal window. | – | User-selected threshold with $0 < f < 1$. |
| $d_{fC_{r,\max}}$ | Distance at which the first-arrival call rate equals $fC_{r,\max}$. | m | Derived terminal-window boundary. |
| $\Delta d_{f\to 1}$ | Spatial interval between $fC_{r,\max}$ and $d_{\mathrm{stop}}$. | m | Derived under the same terminal assumptions. |
| $\Delta t_{f\to 1}$ | Approximate residence time within that terminal spatial interval. | s | Requires a representative, locally constant radial closing velocity. |
| $N_{f\to 1}$ | Expected number of call onsets within the terminal window. | calls | Continuous call-density approximation under constant $v_r$, constant $k_r$, and first-arrival anchoring. |
| $\mathcal{R}_n$, $\widetilde{\mathcal{R}}_n$ | Timing-adjustment metric and its normalisation by $C_{r,\max}$. | Hz, – | Derived sequence diagnostics, not substitutes for $k_r$. |
| $n^*$, $\delta t^*$ | Buzz-readiness call index and the associated absolute change between successive IPIs. | –, s | Model-defined trajectory diagnostics; not physiological reaction-time measurements. |

**Table S2: Simulation and variability notation.** These quantities implement optional numerical constraints or stochastic variability and are not additional components of the core responsivity law.

| Symbol | Definition | Units | Role |
|---|---|---|---|
| $v_{r,n}^{\mathrm{tim}}$ | Non-negative, subsonic radial velocity used only in the simulated acoustic-delay calculation. | m s$^{-1}$ | Numerical timing state; signed $v_r$ is retained for kinematics. |
| $\varepsilon_c$ | Small margin keeping $v_r^{\mathrm{tim}} < c$. | m s$^{-1}$ | Numerical bound. |

*Continued on next page*

*Supplementary Table S2 continued*

| Symbol | Definition | Units | Role |
|---|---|---|---|
| $d_0, v_0$ | Initial anchor distance and initial bat or approach speed specified for a simulated sequence. | m, m s$^{-1}$ | Initial-condition parameters; $v_0$ need not equal eventwise radial closing velocity. |
| $T_b^*$ | Optional minimum behavioural interval. | s | Response-floor parameter. |
| $T_{b,n}^{\text{core}}$ | Core interval after application of the optional response floor. | s | Equals $\max(k_r T_a, T_b^*)$. |
| $T_{b,n}^{\text{eff}}$ | Realised post-acquisition interval after optional caller-side lag. | s | Enters the emitted IPI. |
| $\delta_n, \bar{\delta}_n$ | Realised non-negative caller-side lag and its conditional mean. | s | Optional scheduling variability. |
| $f_\delta, f_{\delta,\max}$ | Mean and maximum lag as fractions of $T_b^{\text{core}}$. | – | Lag-scale parameters. |
| $\epsilon_n$ | Autoregressive deviation of caller-side lag from its mean. | s | Stochastic state. |
| $\rho_\delta$ | Lag-one autocorrelation of $\epsilon_n$. | – | Temporal persistence parameter. |
| $\sigma_{\delta,n}, s_\delta$ | Call-specific innovation standard deviation and its scale relative to mean lag. | s, – | Innovation-magnitude parameters. |
| $\eta_n$ | Independent standard-normal innovation driving caller-side lag. | – | Random variate. |
| $\Delta s_n^{\text{pred}}$ | Timing-predicted radial displacement, $v_r^{\text{tim}} \Delta t_n$. | m | Diagnostic, distinct from realised vector displacement. |
| $T_{c,0}, T_{c,\min}$ | Initial or maximum and minimum permitted call durations. | s | Duration bounds. |
| $g_c$ | Gain linking the next deterministic call duration to the preceding acquisition interval. | – | Causal duration-controller parameter. |
| $T_{c,n}^{\text{det}}, T_{c,n}^{\text{raw}}$ | Deterministic duration before jitter and unconstrained duration after jitter. | s | Intermediate simulation states. |
| $\sigma_c, p_c$ | Additive duration-jitter standard deviation and proportional jitter magnitude. | s, – | Duration-variability parameters. |
| $z_n$ | Standardised additive duration-jitter state. | – | Independent or autoregressive stochastic state. |
| $\rho_c$ | Lag-one autocorrelation coefficient of $z_n$. | – | Duration-jitter persistence. |
| $\xi_n, \zeta_n$ | Independent standard-normal innovations for correlated additive and separate proportional duration jitter. | – | Random variates. |

## S2 CONDITIONAL ANALYTICAL REFERENCE

For compactness, define the propagation-delay component implied by an observed interval under a specified $k_r$ and $\phi$ as

$$A_n \equiv \frac{\Delta t_n}{1 + k_r} - \phi_n T_{c,n} = \frac{1}{(1 + k_r) C_{r,n}} - \phi_n T_{c,n}. \tag{SE1}$$

Under the motion-aware acquisition model, $A_n = 2d_n/(c + v_{r,n})$. A physically admissible positive-range reconstruction therefore requires $A_n > 0$. If $A_n \leq 0$, the selected combination of $k_r$, $\phi$, timing, and call duration is incompatible with a positive propagation delay; assigning zero distance, as in the field diagnostic, is a computational boundary rather than a measurement of zero range.

**Table S3: Analytical lookup table: what can be calculated from which known quantities.** Each row is a conditional calculation, not a claim that the required inputs are ordinarily observable. Equivalent IPI and call-rate forms use $\Delta t = 1/C_r$. Main-text equation references identify the governing derivation.

| Calculated quantity | Quantities that must be known | Conditional expression | Required model level | Principal limitation |
|---|---|---|---|---|
| Acquisition interval $T_a$ from geometry | $d, v_r, \phi, T_c$, anchor identity | $T_a = 2d/(c + v_r) + \phi T_c$ | General motion-aware acquisition model; Equation 19 | The selected anchor and information endpoint must be known. |
| Behavioural interval $T_b$ | $T_a, k_r$ | $T_b = k_r T_a$ | Core proportional law; Equation 25 | Does not identify the internal neural or motor components of $T_b$. |

*Continued on next page*

*Supplementary Table S3 continued*

| Calculated quantity | Quantities that must be known | Conditional expression | Required model level | Principal limitation |
|---|---|---|---|---|
| IPI $\Delta t$ | $d, v_r, \phi, T_c, k_r$ | $\Delta t = (1 + k_r)[2d/(c + v_r) + \phi T_c]$ | General core model; Equation 29 | Excludes an active response floor or unaccounted extra lag. |
| Call rate $C_r$ | $d, v_r, \phi, T_c, k_r$ | Reciprocal of the preceding IPI | General core model; Equation 32 | Same dependencies as IPI; call rate supplies no additional independent equation. |
| $T_a$ from timing | $\Delta t, k_r$, or $C_r, k_r$ | $T_a = \Delta t/(1 + k_r) = 1/[(1 + k_r)C_r]$ | Core proportional law; Equations 33 and 35 | A specified $k_r$ partitions the interval; timing alone does not estimate $k_r$. |
| $T_b$ from timing | $\Delta t, k_r$, or $C_r, k_r$ | $T_b = k_r\Delta t/(1 + k_r) = k_r/[(1 + k_r)C_r]$ | Core proportional law; Equations 34 and 36 | Conditional transformation, not an independent field measurement. |
| Anchor distance $d$, general | $\Delta t$ or $C_r$, $k_r, v_r, \phi, T_c$ | $d = (c + v_r)A/2$ | Motion-aware acquisition model | Requires $A > 0$ and the remaining latent quantities to be independently fixed. |
| Radial velocity $v_r$, general | $\Delta t$ or $C_r$, $k_r, d, \phi, T_c$ | $v_r = 2d/A - c$ | Motion-aware acquisition model | Requires $A > 0$, known anchor distance, and eventwise anchor consistency. |
| Echo-window scale $\phi$ | $\Delta t, k_r, d, v_r, T_c$ | $\phi = [\Delta t/(1 + k_r) - 2d/(c + v_r)]/T_c$ | Motion-aware acquisition model | Requires $T_c > 0$; negative values indicate incompatibility with the adopted non-negative-window interpretation. |
| Responsivity $k_r$, general | $\Delta t, d, v_r, \phi, T_c$ | $k_r = \Delta t/[2d/(c + v_r) + \phi T_c] - 1$ | General core model | All acquisition variables must be independently known; unmodelled lag inflates the result. |
| Anchor distance $d$, first arrival | $C_r, v_r, k_r$ | $d = (c + v_r)/[2(1 + k_r)C_r]$ | $\phi T_c \approx 0$; Equation 39 | Biased if a later echo-window endpoint governs timing. |
| Radial velocity $v_r$, first arrival | $C_r, d, k_r$ | $v_r = 2(1 + k_r)dC_r - c$ | $\phi T_c \approx 0$; Equation 40 | Small timing or distance errors can yield implausible velocities. |
| Call-rate derivative $\dot{C}_r$ | $d, v_r, \dot{v}_r, k_r$ | $\dot{C}_r = \{\dot{v}_r/d + (c + v_r)v_r/d^2\}/[2(1 + k_r)]$ | Stable first-arrival anchor; Equation 41 | Requires differentiable trajectories and locally stable $k_r$. |
| Radial acceleration $\dot{v}_r$ | $d, C_r, v_r, \dot{C}_r, k_r$ | $\dot{v}_r = 2(1 + k_r)d\dot{C}_r - 2(1 + k_r)C_r v_r$ | Stable first-arrival anchor; Equation 42 | Derivatives amplify timing noise. |
| Radial velocity from call-rate trajectory | $C_r(t), \dot{C}_r(t), k_r$ | $v_r \approx c\dot{C}_r/[2(1 + k_r)C_r^2]$ | First arrival, far field, approximately constant $v_r$; Equation 44 | Not an instantaneous call-rate–velocity proportionality; requires smoothing and a stable anchor. |
| Overlap-boundary distance | $T_c, v_r$ | $d_{\text{overlap}} = (c + v_r)T_c/2$ | First-echo overlap condition; Equation 51 | Concerns physical pulse–echo overlap, not the full selected acquisition window. |
| Terminal ceiling $C_{r,\max}$ | $d_{\text{stop}}, v_r, k_r$ | $C_{r,\max} = (c + v_r)/[2(1 + k_r)d_{\text{stop}}]$ | First-arrival terminal reference; Equation 45 | A theoretical ceiling need not be attained in an observed sequence. |
| Responsivity from an attained ceiling | $C_{r,\max}, d_{\text{stop}}, v_r$ | $k_r = (c + v_r)/(2d_{\text{stop}}C_{r,\max}) - 1$ | First arrival and genuinely attained ceiling | Substituting a sub-ceiling observed maximum overestimates $k_r$. |
| Fractional-ceiling distance | $d_{\text{stop}}, f$ | $d_{fC_{r,\max}} = d_{\text{stop}}/f$ | Same $k_r$, $v_r$, and first-arrival rule at both boundaries; Equation 46 | Cancellation fails if those quantities change across the window. |
| Terminal spatial width | $d_{\text{stop}}, f$ | $\Delta d = d_{\text{stop}}(1/f - 1)$ | Same conditions as preceding row; Equation 47 | A model-defined spatial window, not a universal anatomical zone. |
| Terminal residence time | $\Delta d, v_r$ | $\Delta t_{f\to 1} \approx \Delta d/v_r$ | Representative constant $v_r > 0$; Equation 48 | Deceleration requires trajectory integration instead. |

*Continued on next page*

*Supplementary Table S3 continued*

| Calculated quantity | Quantities that must be known | Conditional expression | Required model level | Principal limitation |
|---|---|---|---|---|
| Terminal call number | $f, v_r, k_r$ | $N = (c + v_r)\ln(1/f)/[2(1 + k_r)v_r]$ | Constant $v_r > 0$, constant $k_r$, first arrival; Equation 49 | Continuous update-budget approximation; not an integer event count for a particular sequence. |
| Buzz-readiness index $n^*$ | Complete IPI sequence and specified $C_{r,\max}$ | Minimise $\lvert\mathcal{R}_n - C_{r,\max}\rvert$ | Diagnostic definitions in Equations 53–55 | Model-defined proximity criterion, not a recovered physiological threshold. |

**Table S4: Assumption and identifiability map for the analytical equations.** The rows identify which simplifications permit each family of calculations and what cannot be concluded from them.

| Model level | Assumptions | Permitted use | What remains unresolved |
|---|---|---|---|
| Event decomposition | Correct onset-to-onset IPI and a selected echo-information endpoint. | $\Delta t = T_a + T_b$. | Does not determine either component from timing alone. |
| Proportional responsivity | $T_b = k_r T_a$ over the event or analysed regime; no active floor or unaccounted lag. | Partition IPI into conditional $T_a$ and $T_b$ when $k_r$ is specified. | Does not identify $k_r$, anchor identity, or the internal composition of $T_b$. |
| Motion-aware acquisition | One effective anchor is specified for the interval; local radial motion is represented by $v_r$; selected window is $\phi T_c$. | Forward timing and conditional inversion among $d, v_r, \phi, k_r$. | One timing observation cannot jointly determine these latent quantities. |
| First-arrival special case | $\phi T_c$ is negligible relative to propagation delay. | Simple hyperbolic call-rate identity and Equations 39–40. | Invalid when a later echo feature or extended integration window governs timing. |
| Far-field approximation | First-arrival case and $\lvert v_r\rvert \ll c$. | Replace $c + v_r$ by $c$; derive the approximate timing-based velocity estimator. | Removes the already small instantaneous velocity contribution and is unsuitable for precision inversion without error analysis. |
| Trajectory dynamics | Stable anchor and locally constant $k_r$; differentiable call-rate and motion trajectories. | Relate $\dot{C}_r$, $v_r$, and $\dot{v}_r$. | Switching, irregular sampling, and numerical differentiation can dominate the derivative. |
| Terminal ceiling | First-arrival rule, known $d_{\text{stop}}$, and an actually attained responsivity-defined ceiling. | Infer $C_{r,\max}$ or conditionally rearrange for $k_r$. | An observed maximum below the ceiling supplies no equality estimate of the true limit. |
| Terminal update budget | Constant $v_r > 0$, constant $k_r$, first-arrival timing, and fixed fractional boundary $f$. | Estimate terminal spatial width, residence time, and expected number of updates. | Deceleration, changing anchors, $\phi$, or discrete boundary effects require event-level simulation or integration. |
| Overlap boundary | First relevant echo start is the relevant physical return for pulse–echo overlap. | Infer the distance at which emitted call duration reaches the returning echo. | Does not establish which later echo information closes $T_a$. |
| Simulation with lag or floor | The active constraint and realised lag are known. | Forward simulation; conditional inversion only after explicitly accounting for the added interval. | Naive core inversions attribute floor- or lag-generated time to $T_a$ or $k_r$. |
| Multiple-anchor scene | The effective anchor is established separately for each call interval. | Test each candidate anchor for compatibility with the timing equations. | Beam aim or experimental target identity alone does not prove which echo governed timing. |

## S3 SELECTED DERIVATIONS AND SAFEGUARDS

### S3.1 General inverse family

Combining Equations 29 and SE1 gives $A_n = 2d_n/(c + v_{r,n})$. Consequently, any one of $d_n$, $v_{r,n}$, $\phi_n$, or $k_r$ can be calculated only when the other acquisition variables are independently specified:

$$d_n = \frac{c + v_{r,n}}{2} A_n, \tag{SE2}$$

$$v_{r,n} = \frac{2d_n}{A_n} - c, \tag{SE3}$$

$$\phi_n = \frac{1}{T_{c,n}} \left[ \frac{\Delta t_n}{1 + k_r} - \frac{2d_n}{c + v_{r,n}} \right], \tag{SE4}$$

$$k_r = \frac{\Delta t_n}{2d_n/(c + v_{r,n}) + \phi_n T_{c,n}} - 1. \tag{SE5}$$

These rearrangements are algebraically exact within the core motion-aware model, but they are not jointly identifying. One observed IPI supplies one timing constraint. If several of the anchor distance, radial velocity, echo-window endpoint, and responsivity coefficient are unknown, infinitely many combinations can explain the same interval. Moreover, if a known additional lag $\delta_n$ is present, $\Delta t_n - \delta_n$, rather than $\Delta t_n$, enters the proportional core inversion. When the response floor is active, $T_b \neq k_r T_a$ and this inverse family cannot recover $k_r$ from that event.

### S3.2 First-arrival and terminal rearrangements

When $\phi T_c$ is negligible, $A = \Delta t/(1 + k_r)$ and the general inverse family reduces to Equations 39 and 40. At a known stop distance, rearranging Equation 45 gives

$$k_r = \frac{c + v_r}{2d_{\mathrm{stop}} C_{r,\max}} - 1. \tag{SE6}$$

This is an equality only when the observed rate is the attained terminal ceiling under the assumed anchor and first-arrival rule. If the largest recorded call rate lies below the attainable ceiling, substituting it into Equation SE6 overestimates $k_r$ under otherwise correct assumptions.

For constant radial velocity, the terminal call budget follows by integrating call density per unit distance over the interval from $d_{\mathrm{stop}}$ to $d_{\mathrm{stop}}/f$:

$$N_{f \to 1} = \int_{d_{\mathrm{stop}}}^{d_{\mathrm{stop}}/f} \frac{C_r(d)}{v_r} \, \mathrm{d}d \tag{SE7}$$

$$= \frac{c + v_r}{2(1 + k_r) v_r} \ln\left(\frac{1}{f}\right). \tag{SE8}$$

The cancellation of $d_{\mathrm{stop}}$ in this expression does not make the stopping geometry irrelevant: that distance fixes the physical placement and width of the terminal window. The closed form applies only when $v_r$, $k_r$, and the anchoring rule remain constant across it; otherwise the event-level trajectory must be integrated directly.

### S3.3 Trajectory derivative

For the first-arrival identity $C_r = (c + v_r)/(Kd)$, differentiation with $\dot{d} = -v_r$ gives

$$\dot{C}_r = \frac{1}{K} \left[ \frac{\dot{v}_r}{d} + \frac{(c + v_r) v_r}{d^2} \right]. \tag{SE9}$$

If $\dot{v}_r \approx 0$, substituting $d \approx c/(KC_r)$ in the far field yields Equation 44. Thus, velocity is related to the trajectory derivative of call rate, not directly proportional to instantaneous call rate. Anchor changes or noisy numerical differentiation can violate the conditions needed for this estimator even when individual calls remain compatible with the general timing equation.

## S4 SECONDARY NUMERICAL RESULTS

**Table S5: Echo-window effects on IPI, call duration, and call-rate spread.** Slopes and intercepts are from per-condition fits against anchor distance. Pairwise slopes describe the change in absolute separation between binned median curves per metre; positive values indicate convergence towards the target. For IPI, the omnibus effects of distance, $\phi$, and their interaction were all $p \approx 0$ ($R^2 = 0.997$); for call duration, the corresponding model had $R^2 = 0.855$. In the call-rate bend window, mean IQRs were 8.80, 7.81, and 7.28 Hz for $\phi = 0, 0.5$, and 1; neither $\phi$ ($p = 0.890$) nor its interaction with distance ($p = 0.955$) explained spread.

| Response | $\phi$ | $n$ | Slope (ms m$^{-1}$) | Intercept (ms) | $R^2$ |
|---|---|---|---|---|---|
| IPI | 0 | 1588 | 34.36 | 0.02 | 1.000 |
| IPI | 0.5 | 1340 | 37.62 | 1.64 | 0.999 |
| IPI | 1 | 1111 | 40.42 | 5.62 | 0.994 |
| Call duration | 0 | 1588 | 1.09 | 0.33 | 0.902 |
| Call duration | 0.5 | 1340 | 1.08 | 0.55 | 0.852 |
| Call duration | 1 | 1111 | 1.01 | 0.94 | 0.785 |
| **Response** | **$\phi$ pair** | **Bins** | **Separation slope** | **$p$** | **$R^2$** |
| IPI | 0 versus 0.5 | 17 | 3.31 | $2.24 \times 10^{-8}$ | 0.883 |
| IPI | 0 versus 1 | 17 | 6.14 | $2.32 \times 10^{-7}$ | 0.840 |
| IPI | 0.5 versus 1 | 17 | 2.83 | $1.73 \times 10^{-5}$ | 0.719 |
| Call duration | 0 versus 0.5 | 17 | 0.014 | 0.767 | 0.006 |
| Call duration | 0 versus 1 | 17 | 0.096 | 0.237 | 0.092 |
| Call duration | 0.5 versus 1 | 17 | 0.085 | 0.054 | 0.225 |

**Table S6: Interval–distance fits for the responsivity, echo-window, and velocity sweeps.** Slopes and intercepts are given separately for $T_a$ and $T_b$. The nested interaction tests detected distance, condition, and distance-by-condition effects in every sweep ($p \approx 0$); the near-identical $T_a$ slopes across $k_r$, and the small changes across velocity, show why effect magnitude rather than significance is decisive here. For the $\phi = 0$ versus 1 curves, binned separation increased with distance at 0.48 ms m$^{-1}$ for $T_a$ and 2.38 ms m$^{-1}$ for $T_b$ ($p < 0.001$).

| Sweep | Level | $n$ | $T_a$ slope | $T_a$ intercept | $T_b$ slope | $T_b$ intercept | $R^2$ |
|---|---|---|---|---|---|---|---|
| $k_r$ | 3 | 1867 | 5.99 | 0.65 | 17.96 | 1.94 | 0.998 |
| $k_r$ | 5 | 1283 | 6.05 | 0.37 | 30.23 | 1.85 | 0.999 |
| $k_r$ | 7 | 955 | 6.05 | 0.25 | 42.38 | 1.74 | 0.999 |
| $k_r$ | 9 | 759 | 6.05 | 0.19 | 54.43 | 1.73 | 0.999 |
| $\phi$ | 0 | 1433 | 5.74 | 0.00 | 28.72 | 0.01 | 1.000 |
| $\phi$ | 0.5 | 1274 | 6.04 | 0.37 | 30.22 | 1.85 | 0.999 |
| $\phi$ | 1 | 1106 | 6.29 | 0.97 | 31.44 | 4.83 | 0.995 |
| $v_0$ (m s$^{-1}$) | 2 | 3292 | 6.13 | 0.29 | 30.64 | 1.46 | 0.999 |
| $v_0$ (m s$^{-1}$) | 4 | 1624 | 6.07 | 0.36 | 30.35 | 1.78 | 0.999 |
| $v_0$ (m s$^{-1}$) | 6 | 1042 | 6.02 | 0.39 | 30.08 | 1.96 | 0.999 |
| $v_0$ (m s$^{-1}$) | 8 | 762 | 5.96 | 0.49 | 29.79 | 2.44 | 0.999 |
| $v_0$ (m s$^{-1}$) | 10 | 602 | 5.91 | 0.53 | 29.54 | 2.65 | 0.999 |

**Table S7: Secondary statistics for the field–simulation comparison and conditional profiles.** The first three rows compare measured quantities; subsequent rows are conditional transformations or profile diagnostics under fixed $k_r = 5$ and the stated simulation geometry. They must not be interpreted as independent measurements of target distance, $T_b$, or $\phi$. The initial-distance sensitivity sweep covered $d_0 = 2$–8 m; both diagnostics retained the global minimum at $d_0 = 5$ m and $\phi = 1.5$ on the coarser grid, although the preferred $\phi$ varied with $d_0$.

| Quantity | Field | Simulation | Comparison or profile result |
|---|---|---|---|
| Effective velocity (m s$^{-1}$) | 6.461 (5.239–7.593) | 6.460 (5.209–7.606) | Velocity-band occupancy: $\chi^2 = 0.011$, $df = 4$, $p = 0.999985$ |
| IPI interaction model | 4425 calls | 4427 calls | Dataset-dependent terms $p \approx 0$; $R^2 = 0.969$ |
| Duration-envelope compatibility | 20 seeded ensembles | | Median 94.1% (IQR 93.1–95.0%) |
| Distance profile | 1.668 m (1.074–1.946) | 1.259 m (0.727–1.983) | Minimum at $\phi = 1.35$; mean quantile gap 0.362 m; 1.79% clipped at zero |
| $T_b$ profile | 68.59 ms (50.05–77.03) | 49.99 ms (32.09–74.83) | Minimum at $\phi = 1.20$; mean quantile gap 16.83 ms |
| Rank-matched required $\phi$ | Median 1.76 (IQR 1.05–2.22) | | Conditional diagnostic, not parameter recovery |
| Initial-distance sensitivity | Candidate $d_0 = 2$–8 m | | Global minimum retained at $d_0 = 5$ m; best $\phi = 1.5$ on coarse grid |

**Table S8: Buzz-readiness summaries across responsivity, velocity, and initial call duration.** Values are grouped medians; parentheses give the interquartile range for readiness distance. Each $k_r$ level contains 400 sequences, each velocity level 320 sequences, and each duration level 400 sequences.

| Sweep | Level | Readiness distance (m) | $\delta t^*$ (ms) | Calls per sequence |
|---|---|---|---|---|
| $k_r$ | 3 | 1.98 (1.43–3.16) | 3.52 | 47.5 |
| $k_r$ | 5 | 1.37 (1.01–2.00) | 5.43 | 32 |
| $k_r$ | 7 | 1.02 (0.70–1.45) | 6.78 | 23 |
| $k_r$ | 9 | 0.80 (0.52–1.19) | 8.72 | 19 |
| $v_0$ (m s$^{-1}$) | 1 | 3.55 (2.75–4.54) | 6.10 | 85 |
| $v_0$ (m s$^{-1}$) | 2 | 1.72 (1.32–2.49) | 6.00 | 42 |
| $v_0$ (m s$^{-1}$) | 3 | 1.19 (0.91–1.67) | 6.37 | 27.5 |
| $v_0$ (m s$^{-1}$) | 4 | 0.85 (0.61–1.22) | 6.12 | 20 |
| $v_0$ (m s$^{-1}$) | 5 | 0.66 (0.52–0.98) | 5.96 | 16 |
| $T_{c,0}$ (ms) | 3 | 1.29 (0.80–2.25) | 6.01 | 30 |
| $T_{c,0}$ (ms) | 5 | 1.28 (0.80–2.24) | 5.98 | 30 |
| $T_{c,0}$ (ms) | 7 | 1.28 (0.80–2.24) | 5.97 | 30 |
| $T_{c,0}$ (ms) | 10 | 1.28 (0.79–2.24) | 5.96 | 30 |

**Table S9: Proxy and time-to-contact (TTC) comparisons for buzz readiness.** The $\delta t^* - T_{b,\mathrm{min}}$ columns apply to the $k_r$ sweep. TTC columns compare the median remaining time from readiness with the time from $d_{\mathrm{stop}}$. Paired tests for all TTC comparisons gave $p \approx 0$ and 0% agreement within 10%.

| Sweep | Level | $\delta t^* - T_{b,\mathrm{min}}$ (ms) | $p$ | Within 10% | Readiness TTC (ms) | $d_{\mathrm{stop}}$ TTC (ms) | $d_z$ |
|---|---|---|---|---|---|---|---|
| $k_r$ | 3 | 0.599 | $\approx 0$ | 20% | 660 | 50 | 0.86 |
| $k_r$ | 5 | 0.495 | $\approx 0$ | 35% | 458 | 50 | 0.78 |
| $k_r$ | 7 | −0.045 | 0.484 | 100% | 338 | 50 | 0.76 |
| $k_r$ | 9 | −0.419 | 0.0043 | 80% | 266 | 50 | 0.75 |
| $v_0$ (m s$^{-1}$) | 1 | – | – | – | 3554 | 150 | 3.67 |
| $v_0$ (m s$^{-1}$) | 2 | – | – | – | 861 | 75 | 2.45 |
| $v_0$ (m s$^{-1}$) | 3 | – | – | – | 398 | 50 | 2.54 |
| $v_0$ (m s$^{-1}$) | 4 | – | – | – | 213 | 37.5 | 2.20 |
| $v_0$ (m s$^{-1}$) | 5 | – | – | – | 132 | 30 | 1.96 |

**Table S10: Structure of SSG-like events in the productive multi-target conditions.** Stable IPI and anchor distance are means with SD and interquartile range. Group-size counts are given as doublets/triplets/quadruplets.

| Condition | Events | Group sizes | Stable IPI (ms) | Anchor distance (m) | Same anchor |
|---|---|---|---|---|---|
| C3: stationary, random anchor | 78 | 77/1/0 | 10.30 (5.30; 7.27–11.80) | 0.296 (0.152; 0.209–0.339) | 100% |
| C6: moving, random anchor | 438 | 405/32/1 | 13.14 (7.14; 8.78–14.89) | 0.378 (0.205; 0.252–0.427) | 98.4% |
